# Importance of a Follow-Up Ultrasound Protocol in Monitoring Posttraumatic Spleen Complications in Children Treated with a Non-Operative Management

**DOI:** 10.3390/medicina57080734

**Published:** 2021-07-21

**Authors:** Ivona Djordjevic, Dragoljub Zivanovic, Ivana Budic, Ana Kostic, Danijela Djeric

**Affiliations:** 1 Pediatric Surgery Clinic, Clinical Center, 18000 Nis, Serbia; ivona.djordjevic@medfak.ni.ac.rs (D.Z.); anakosticmd@gmail.com (A.K.); danijeladjericmd@gmail.com (D.D.); 2 Faculty of Medicine, University of Nis, 18000 Nis, Serbia; md.ivanabudic@gmail.com; 3 Clinic for Anestesiology and Intensive Therapy, Clinical Center Nis, 18000 Nis, Serbia

**Keywords:** posttraumatic spleen complications follow-up

## Abstract

*Background and objectives*: For the last three decades, non-operative management (NOM) has been the standard in the treatment of clinically stable patients with blunt spleen injury, with a success rate of up to 95%. However, there are no prospective issues in the literature dealing with the incidence and type of splenic complications after NOM. *Materials and methods*: This study analyzed 76 pediatric patients, up to the age of 18, with blunt splenic injury who were treated non-operatively. All patients were included in a posttraumatic follow-up protocol with ultrasound examinations 4 and 12 weeks after injury. *Results*: The mean age of the children was 9.58 ± 3.97 years (range 1.98 to 17.75 years), with no statistically significant difference between the genders. The severity of the injury was determined according to the American Association for Surgery of Trauma (AAST) classification: 7 patients had grade I injuries (89.21%), 21 patients had grade II injuries (27.63%), 33 patients had grade III injuries (43.42%), and 15 patients had grade IV injuries (19.73%). The majority of the injuries were so-called high-energy ones, which were recorded in 45 patients (59.21%). According to a previously created posttraumatic follow-up protocol, complications were detected in 16 patients (21.05%). Hematomas had the highest incidence and were detected in 11 patients (14.47%), while pseudocysts were detected in 3 (3.94%), and a splenic abscess and pseudoaneurysm were detected in 1 patient (1.31%), respectively. The complications were in a direct correlation with injury grade: seven occurred in patients with grade IV injuries (9.21%), five occurred in children with grade III injuries (6.57%), three occurred in patients with grade II injuries (3.94%), and one occurred in a patient with a grade I injury (1.31%). *Conclusion*: Based on the severity of the spleen injury, it is difficult to predict the further course of developing complications, but complications are more common in high-grade injuries. The implementation of a follow-up ultrasound protocol is mandatory in all patients with NOM of spleen injuries for the early detection of potentially dangerous and fatal complications.

## 1. Introduction

In spite of all the preventive measures taken, trauma remains the leading cause of death and incapacity of children in the modern world [1]. The spleen is the most commonly injured solid abdominal organ in blunt abdominal trauma (BAT), accounting for up to 46% of all abdominal parenchymal lesions [2,3].

Conservative, non-operative management (NOM) has been, for the last three decades, the standard in the treatment of most clinically stable patients with blunt spleen injury [4], with a success rate of up to 95% [5,6]. It can only be performed in hemodynamically stable patients on continuous monitoring in the intensive care unit, with an experienced multidisciplinary team.

The treatment method of splenic injuries is multifactorial depending on age, hemodynamic stability, injury grade, and comorbidity, and older adult patients have smaller chances for successful NOM [7]. The splenic capsule is more compact in the children than in adults (with a higher number of myofibroblasts and collagen fibers), resulting in the retention of bleeding under the capsula and increasing the possibility of NOM. Smaller circulation volume in children (80 mL/kg) is the reason for rapid heat loss. Paradoxically, despite extreme hypovolemia, blood pressure is maintained due to the good adaptability of the compensation mechanism of the injured child, and secondary hypovolemic hypotension is late. This great hemostatic capability is an advance in treating children with NOM [8].

Multi-slice computer tomography (MSCT ) with intravenous contrast is now considered as imperative for grading the solid abdominal organs injuries according to the criteria established by American Association for Surgery of Trauma (AAST). It detects bleeding from the injured organ, with sensitivity of 92–97.6% and a specificity of 98.7%.

Insistence on spleen preservation is based on its significant immune role as well as the prevention of potentially fatal postsplenectomy sepsis (PSS).

According to the literature, to date, there is no well-defined time required for complete spleen healing after BAT. CT findings indicate that 90% of grade III injuries heal by 76 ± 7 days, while 77% of grade IV injuries heal within 81 ± 8 days [9]. Possible complications should be occur during this time interval, but most commonly, they occur in the first half of this period. As there are no specific biomarkers in the monitoring of spleen function in the initial and posttraumatic period, abdominal echo sonography is considered to be the “first choice” diagnostic tool to be performed in regular time intervals with an MSCT/NMR for unclear cases or for additional, more precise, findings.

There are few non-prospective studies that tried to prove the justification of NOM correlated with the rate of development of posttraumatic complications, especially in children. Therefore, the idea behind this study was to create a protocol for posttraumatic monitoring of patients with a spleen injury, which includes an abdominal ultrasound examination to determine the incidence of complications after NOM of blunt spleen injuries.

## 2. Material and Methods

The patients were admitted and treated in our institution from 1 January 2016 to 31 December 2020 and were monitored prospectively. The inclusion criteria were hemodynamically stable patients with isolated splenic injury caused by blunt abdominal trauma. All patients with systolic blood pressure above 80 mmHg, or those stabilized after a blood transfusion (less than 40 mL/kg), with no signs of peritoneal irritation, were considered stable and included in the study. The exclusion criteria were hemodynamically unstable patients, and patients with multiple organ injuries or patients treated surgically. Injury grades were classified according to the recommendations of the AAST, which defined the most comprehensive classification of abdominal injuries, revised in 1994, and based on MSCT diagnostics [10]. Although the extravagation of contrasts indicates a higher blood vessels injury, which imposes the need for laparotomy, it does not have to be a rule for exclusion criteria in children.

## 3. Data Collection

After institutional review board approval (by the Ethics Committee of Faculty of Medicine Niš No 01-10204-1, from 15 December 2015), data were collected from hospital charts. Informed consent for every child included in the study was signed by their parents, in accordance with the recommendations of the World Medical Association Declaration of Helsinki—Ethical principles for medical research involving a human subject [11].

The patients were either admitted to our clinic through the Emergency Service, referred from regional secondary centers, or transferred after unsuccessful initial treatment in other regional institutions. Data collected from data charts included demographics such as age and gender. Clinical data obtained included mechanism of injury, injury grade, hemoglobin (mmol/L), and complications (if any). 

All ultrasound examinations were performed on an ultrasonic device, the color Doppler ACUSON X 300 (Siemens, Erlangen, Germany), which uses convex and linear sondes of a frequency of 2–10 MHz, with the realization of longitudinal, transverse, oblique scans, and B mode scans.

The abdominal organs were examined by an MSCT, performed at the Radiology Center. The MSCT was performed with an scanner Aquilion 64 (Toshiba, Tokyo, Japan), according to the standard abdominal protocol: with native and contrast scans (the arterial and parenchymal phase), and an additional 3D reconstruction [12]. The results were interpreted by experienced radiologists and recorded on a CD.

For additional and more precise data, in complicated cases, diagnosis was completed with Avanto 1.5T MAGNETOM (Siemens, Erlangen, Germany), according to standard 3-phase abdominal protocols: native, contrast, and dynamic. T1w/T2W IR/true FISP coronary and T1w/t1w FS/T2w FS/t2w/true FISP axial MR abdominal tomograms, as well as postcontrast T1w tomograms, with a cross-section thickness of 5 mm and a space of 0.5 mm cross-sections were used.

## 4. Statistical Analysis

A unique database was created in an MS Office Excel (Microsoft, St.Redmond, WA, USA) spreadsheet, while the SSPS software package (IBM, New York, NY, USA) was used for further processing. Continuous variables are expressed as the means ± standard deviation (SD). Categorical variables were expressed as simple values and percentages (*n*, %). Student’s *t*-test, ANOVA, Chi-square, and Fisher’s test were used for comparisons, and *p* values of ≤0.05 were considered statically significant. 

## 5. The Follow-Up Protocol

The main goal was to a create posttraumatic follow-up abdominal ultrasonography protocol for all patients with spleen injury treated with NOM.

Patients with low-grade injuries (I–III) were discharged from hospital after one to three weeks, but the discharging of patients with high grade injuries (IV–V) was decided based on by their clinical condition. Three months after trauma was the crucial period for the onset of potentially dangerous complications. There were two critical periods in this time interval. The period of up to one month after injury was critical for detecting early complications, and the period three months after trauma was critical for detecting late potentially dangerous complications. So, we established a mandatory posttraumatic protocol that includes at least two ultrasound examinations, 4 and 12 weeks after trauma. Based on these facts, all patients were scheduled for follow-up ultrasound examinations 4 weeks and 3 months (12 weeks) after discharge. In case of complications and unclear cases, patients were scheduled for an NMR/MSCT, but this was not a standard part of the protocol. Complications were treated conservatively or surgically, depending on the symptoms and severeness. 

## 6. Results

This study analyzed 76 pediatric patients who met the inclusion criteria: 50 boys (65.8%) and 26 girls (34.2%), up to the age of 18, with blunt splenic injury who were treated non-operatively. The prospective study analyzed all hemodynamically stable patients with BAT of the spleen admitted and treated in the Pediatric Surgery Clinic during the 5-year interval (from January 2016 to December 2020). 

The mean age of the children was 9.58 ± 3.97 years (range 1.98 to 17.75 years), with no statistically significant difference between the genders (r = 0.706). The highest incidence of injuries was between the ages of 9 and 11. A detailed overview of the patients’ demographics and distribution of splenic injuries in the age groups is summarized in Table 1.

**Figure 1:**
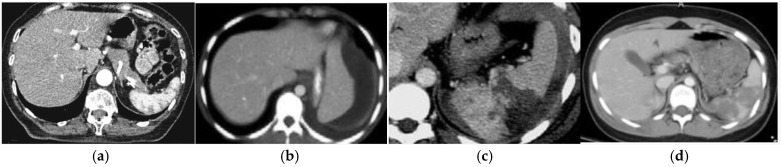
(**a**) Grade I spleen injury, small lacerations as a result of sport injury; (**b**) grade II splenic injury (hematoma) developed after fall from a standing height; (**c**) grade III injury as a consequence of a car accident (splenic lacerations); (**d**) grade IV splenic injury, result of fall from a bicycle.

The severity of the injury was determined according to the AAST classification: 7 patients had grade I injuries (9.21%), 21 had grade II injuries (27.63%), 33 had grade III injuries (43.42%), and 15 patients had grade IV injuries (19.73%). Figure 1 shows the differences in ultrasound findings depending on the AAST classification. 

One patient was excluded from the study. A 12-year-old-boy, injured in a car accident, was admitted to the ICU with no signs of peritoneal tenderness, with regular blood pressure of 110/70mm Hg and an AAST grade IV injury. The initial hemoglobin level was 10 g/dL, and he was included in the study. During the next six hours, massive bleeding and hemorrhagic shock occurred. Despite a transfusion of three units of red blood cells, the levels of hemoglobin remained low (7g/dL), blood pressure was 60/40 mmHg, the abdomen become distended, and an ultrasound examination revealed an extensive amount of free fluid. The patient was referred for surgery and a total splenectomy was performed. The patient was subsequently excluded from the study after surgery. 

The majority of injuries were so-called high-energy ones (motor vehicle injury, fall from a height. The most common cause of BAT was a fall from a height (>1 m or a standing height). The distribution of the splenic injury grade (according to the AAST classification) and trauma mechanisms is presented in Table 2.

All patients were scheduled for a follow-up ultrasound examination 4–12 weeks after discharge.

A posttraumatic control echo sonography examination detected complications in 16 patients (21.05%) (a non-resorbed hematoma, pseudocyst, abscess, and splenic pseudoaneurysm). Although the hematoma was not really a complication but a stage in spleen healing, in a broad sense, it represented a potential complication because it could lead to the development of splenic abscesses and sepsis in case of bacterial superinfection. Hematomas had the highest incidence, and they were detected in 11 patients (14.47%), while pseudocysts were detected in 3 patients (3.94%) and a splenic abscess and pseudoaneurysm were detected in 1 patient (1.31%), respectively. 

**Figure 2:**
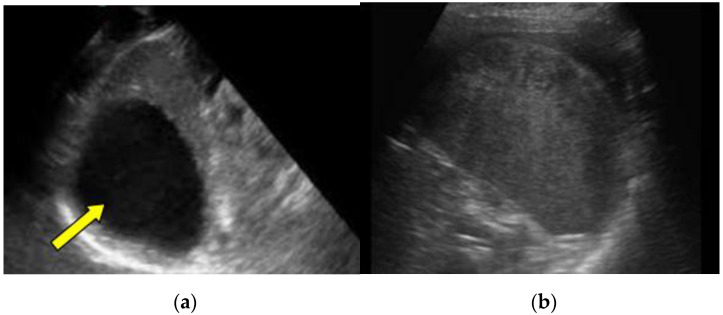
(**a**) A follow-up ultrasound examination, three months after the grade IV splenic injury (patient No. 16 in Table 3). Oblique ultrasound scans diagnosed regularly marginated hypoechoic pseudocyst measuring 50 × 55 mm^2^ in size; (**b**) A transverse scans on a follow-up ultrasound examination, 30 days after trauma revealed subcapsular hematoma 52 × 23 mm^2^ in size. Patient had grade III splenic injury after a fall from a standing height (patient No. 3 in Table 3).

The complications were in direct correlation with the injury grade: seven were found in patients with grade IV injuries (9.21%), five were found in children with grade III injuries (6.57%), three were found in patients with grade II injuries (3.94%), and one was found in a patient with a grade I injury (1.31%). Figure 2 presents marginated hypoechoic pseudocyst after the grade IV splenic injury (2a) and subcapsular hematoma after the grade III splenic injury (2b). 

Complications were detected predominantly in male patients 62.5% (male to female ratio 10:6), with a median age 10.43 years (Table 3). All complications were detected on a follow-up examination. We found three patients with a pseudocyst that formed after BAT of the spleen. Two of them were asymptomatic, and cysts were detected during a scheduled follow-up ultrasound examination. The evolution of the pseudocyst was favorable, with spontaneous regression observed in both patients. In the case of the third patient with a grade IV injury, a large posttraumatic cyst was detected one month after injury, and it continued to grow (50 × 55 mm^2^) during the follow-up, causing pain. The surgical team decided for a partial splenectomy. In a study, we reported a 13-year-old male patient with a grade III spleen injury (a subcapsular hematoma greater that 50% of the surface) that occurred during a sports activity. He was hemodynamically stable, and the clinical outcome was favorable, so he was discharged from hospital after 17 days. After one month, the patient developed a fever of 39 °C accompanied by left lower chest pain. An ultrasound follow-up examination revealed a hypo-echogenic subcapsular liquid collection (32 × 53 mm^2^), which was suitable for the pus collection. After broad spectrum antibiotics administration, the abscess did not regress, and punction was done. A detailed overview of the patients’ demographics, injury grade, cause of the injury, complication characteristics, and treatment are summarized in Table 3.

## 7. Discussion

The spleen is the most frequently injured solid organ in BAT, followed by the liver, kidney, and pancreas [13,14,15]. Introduction of conservative, non-operative management (NOM) in BAT of the spleen is a major advancement in the history of pediatric surgery trauma. It was first implemented by Upadhyaya et al., who published on the conservative treatment of splenic trauma in children at the Sick Children’s Hospital in Toronto in 1968 [4]. The first large multicenter study of 1818 pediatric patients with the success of NOM in 1729 patients (98.56%) with blunt splenic trauma was reported by Holmes et al. [16]. Hashemzadeh et al. reported similar results, noting the success of NOM in 94.1% of spleen injuries, claiming a direct correlation between the severity of injury and failure of NOM [17]. Finally, based on a review of 196 references in the literature that have dealt extensively with the treatment of spleen injuries, following NOM guidelines, as recommended by the Eastern Association for the Surgery of Trauma, Stassen et al. concludes: NOM of blunt spleen injuries is the treatment of choice in all hemodynamically stable patients regardless of age and the severity of the injury, and it should be implemented whenever continuous monitoring, serial clinical examinations, and a surgical room for a possible urgent laparotomy are available [18]. 

Preserving the spleen should be an imperative mostly to prevent postsplenectomic sepsis (PSS). PSS is a well-described potentially very fatal infection, but illness is more severe and even fulminant with mortality rate up to 80%, especially in the younger population. The spleen is an organ with strong immune capability. The main role of the spleen is the capacity to filter and phagocyte infective agents from the blood and production of the opsonins. Absence of the spleen is great attack on young organism, creating a favorable “field” for potentially fatal infection by pneumococci, staphylococci, and haemophilus influenzae. Therefore, the spleen preservation is more beneficial than treating possible complications after NOM of the splenic trauma. Selective splenic angioembolization is a very useful and relatively safe minimal invasive therapeutic procedure in the adults and adolescents for preserving the splenic tissue. Unfortunately, there are some limitations of this method in the younger children. The challenges are related to the smaller size of the splenic arteries, which requires very sophisticated equipment and an experienced radiologist with only a few reports in the literature about this procedure in young patients [19]. 

The introduction of a conservative approach in the treatment of blunt spleen injuries resulted in the occurrence of potentially dangerous, posttraumatic splenic complications (subcapsular and intraparenchymal hematomas, pseudocysts, splenic and subphrenic abscesses, and splenic vascular malformations). The incidence of these complications after splenic injuries in adults according to the literature data is up to 17.6% [20]. There are almost no issues in the literature dealing with the incidence of posttraumatic splenic complications after NOM in the population of children. The aim of the study was to point out the importance of continuous follow-up in all patients treated with NOM. In our study, sixteen patients (21.05%) had delayed complications, including eleven patients with intraparenchymal hematomas, three with pseudocysts, and one with a splenic pseudoaneurysm (SPA) and splenic abscess. 

Most splenic injuries manifest immediately after the trauma as hypovolemic shock and intraperitoneal hemorrhage. Delayed rupture of the spleen occurs very rarely in the pediatric population. It is a result of ruptured subcapsular hematoma. The incidence is very low because of specific splenic capsula composition, which contains a lot of fibromuscular fibers [4]. However, the possibility of a subsequent rupture of the splenic hematoma exists and must not be anticipated. Rupture usually occurs a few days after the initial trauma, when the intrahematoma pressure increases over capsula’s elasticity, causing so-called secondary bleeding. Sometimes, it could be fatal. Fortunately, most hematomas solve without rupture. The most frequent complication of hematomas is infection, which could be seen even a few months after trauma. Preferred treatment is the administration of antibiotics. Punctation is a therapeutic option in case of NOM failure.

Splenic pseudocysts occur with an incidence up to 0.4% [21,22]. As indicated by existing data, in 60% of patients, pseudocysts remain asymptomatic until causing the pain by expansive growth, mechanical pressure, or superinfection [23]. It is generally accepted that cysts smaller than 5 cm will spontaneously regress, but larger growing cysts require surgical intervention due to the threat of rupture [24]. Although surgical options vary from percutaneous drainage, fenestration, and cystectomy to total or partial splenectomy, the preservation of splenic tissue is imperative in the pediatric age. In our study, percutaneous drainage was certainly possible, but the study disregarded it because of the very high incidence of recurrence [25]. A better option for treatment was the partial splenectomy, avoiding a total splenectomy and preventing potentially fatal PSS. 

Splenic abscesses are caused by a splenic contusion and infection of the central or subcapsular hematoma, which occurs in less than 2% of patients and usually remains undetected. After trauma, there is often a latent period between the injury and the clinical presentation that is usually longer than two weeks and even several months [26]. According to the literature, treatment options vary from antibiotic treatment, percutaneous aspiration, and drainage to a partial or total splenectomy. We continued to follow the initially postulated treatment to be conservative. Our management consisted of intravenous broad-spectrum antibiotic therapy and then percutaneous aspiration as an alternative in case of failure. In adults, conservative treatment is rarely successful mostly due to immunodeficiency and chronic diseases. On the contrary, a posttraumatic abscess in a pre-healthy non-immunocompromised child has a high rate of healing, up to 98% [27].

A splenic pseudoaneurysm (SPA) is a possible fatal sequela of BAT. It is commonly asymptomatic and may disappear spontaneously over time. It is a potentially very dangerous complication causing secondary hemorrhaging and hypovolemic shock. In fact, the true incidence of SPA is unknown due to a lack of sufficient comparative patient controls after successful NOM of blunt splenic trauma, especially in the pediatric population. The incidence of SPA is estimated to be around 13%, as Yerdeni et al. reported [28]. Clinical manifestations may vary from abdominal pain to mute (asymptomatic) cases, as it was reported by one girl in our study. The value of the Doppler ultrasound in the diagnosis of a pseudoaneurysm is well documented in the literature, especially for early diagnosis [29]. Certainly, the most accurate diagnostic tool is a contrast-enhanced CT scan, which is much more informative and precise. This was the principle that we followed in the study. SPA is extremely rare, with only eleven cases published in the English literature. Even the American Pediatric Surgical Association Trauma Committee (APSATC) does not recommend routine follow-up contrast CT scans for the detection of PSA, regardless of the injury grade. In a retrospective study in children, Safavi et al. found SPA in 8% of children with grade III splenic injuries and 17% of children with grade IV splenic injuries using a Doppler ultrasound [30]. 

In our study, a seven-year-old girl, after a grade IV motor vehicle injury, was scheduled for a follow-up ultrasound examination on the 32nd day. The girl had no specific symptoms or signs of illness. An experienced radiologist performed a Doppler ultrasound examination, detected SPA, and suggested a contrast-enhanced CT scan for more precise diagnosis. The CT confirmed the diagnosis of SPA with an arteriovenous(AV) fistula. The management of SPA can be operative or non-operative, using endovascular techniques (angioembolization). However, in children, there are no strong evidence-based guidelines for the management of SPA. According to the recommendations for adults, we established a principle of conservative treatment of SPA based on continuous monitoring and angioembolization in case of failure of spontaneous regression. Our patient was monitored by a Doppler ultrasound examination in a 15 to 20-day interval, and during a period of four months, PSA spontaneously evolved.

The spontaneous evolution of SPA in children is very possible due to the self-tamponade effect. Splenic tissue in children is characterized by a high percentage of myoepithelial cells coexisting with higher elasticity of the parenchyma and the ability of splenic arteriola contraction [4]. The bleeding process is self-limited, and a high percentage of spontaneous evolution can be expected. Although the APSATC does not recommend routine follow-up because of low risk of posttraumatic SPA, we recommend it as mandatory in all cases, especially in high-grade injuries.

The significance of this prospective study was in creating a mandatory follow-up ultrasound examination for monitoring the possible complications in a 4–12-week interval after BAT based on the report of Kerpetis et al. The authors concluded that the critical time frame for the development of possible complications is 1 to 3 months after trauma when injury scarification occurs [31]. The scars are thought to be the result of the fibroblast activity that occurs in the field of smaller lacerations. According to our recommendations, it is necessary to carry out control examinations 4–12 weeks after the injury in order for the results to be realistic. This is the time frame for fibroblast mobilization, when complete healing of lesions occurs, and if complications develop, they will generally occur during this interval [32]. Although only an ultrasound examination is considered sufficient for a follow-up, there are some suggestions in the literature that an ultrasound, due to its lack of sensitivity, should be the initial step for detecting possible complications, while the MSCT and/or NMR are needed for more precise information. Most complications are initially asymptomatic, and a routine follow-up examination is very important for their early detection, tracking, and the prevention of potentially fatal conditions. Control ultrasounds were performed over a time interval of one to three months from date of injury to avoid unnecessary radiation. Numerous studies give priority to an NMR examination rather than an ultrasound because it is much more precise and objective. Although there is no radiation during the NMR procedure, it is not widely used because of its cost. In our study, an ultrasound examination was mandatory in all patients, but the NMR and/or MSCT was an alternative additional diagnostic procedure only in cases where an ultrasound examination was not precise enough. A posttraumatic hematoma was found in sixteen patients (21.05%). Hematomas were an “area” where spleen complications may develop and would remain potentially unrecognized in the absence of clinical symptoms and signs. Goleti et al. recommend follow-up US and US color Dopplers for all patients to prevent posttraumatic hematoma complications [33]. In a study of 228 patients, reported by Kristoffersen and Mooney, the incidence of splenic pseudocysts after BAT is very low, and it is found in only one patient (0.44%). They concluded that the number of children with possible complications would probably be even greater if they had undergone routine ultrasound and NMR examinations as well [22].

The incidence of all kinds of complications accompanying NOM spleen injuries in published studies varies, but it does not exceed 7.5% [34]. The incidence of complications after BAT of the spleen in children may have been higher, but follow-up lasted up to six months after the injury, because the highest complication rate was expected in this time frame. Some complications may occur after a few months or even years, but their percentage is much lower, and we believe it does not significantly affect the percentage of complications.

## 8. Conclusions

Based on the severity of the spleen injury, it is difficult to predict the further course of developing complications. There is no obvious correlation between the anatomical severity of the injury and the clinical course, but complications are more common in high-grade injuries (gr III–IV). Implementation of a follow-up ultrasound protocol is mandatory in all patients with NOM of spleen injuries. A higher percentage of complications can be found up to three months after the injury, rendering it the time frame for follow-up ultrasound examinations.

## Figures and Tables

**Table 1:** Patient demographics, and age groups distribution of splenic injuries.

	Gender		Χ^2^ Test	P
**Age** **Groups**	***N***			
	**M**	**F**	**3.601**	**0.706**
**0–2**	**1**	**0**		
**3–5**	**6**	**2**		
**6–8**	**7**	**10**		
**9–11**	**16**	**8**		
**12–14**	**13**	**4**		
**15–17**	**7**	**2**		
**Ʃ**	**50**	**26**		
**X ± SD**	**9.76 ± 4.47**	**9.25 ± 2.90**		

**Table 2:** The distribution of splenic injury grade (AAST classification) according to mechanism of trauma.

Trauma Mechanism	*N* (%)	Grade I	Grade II	Grade III	Grade IV
		***N* (%)**	***N* (%)**	***N* (%)**	***N* (%)**
Motor vehicle injury	16 (21.05%)	1 (1.31%)	3 (3.93%)	9 (11.79%)	3 (3.93%)
Fall from a standing height	20 (26.31%)	2 (2.62%)	6 (7.86%)	9 (11.79%)	3 (3.93%)
Fall from a height > 1 m	24 (31.57%)	2 (2.62%)	9 (11.79%)	8 (10.48%)	5 (6.55%)
Fall from a bicycle	5 (6.57%)	0 (0%)	1 (1.31%)	2 (2.62%)	2 (2.62%)
Sport injury	4 (5. 26%)	1 (1.31%)	1 (1.31%)	2 (2.62%)	0 (0%)
Fight injury	7 (9.21%)	1 (1.31%)	1 (1.31%)	3 (3.93%)	2 (2.62%)
Ʃ	76 (100%)	7 (9.21%)	21 (27.63%)	33 (42.42%)	15 (19.73%)

**Table 3:** Details of various complications in patients treated with NOM after blunt trauma of the spleen.

Demographics, Injury Grade, and Cause							
Patient N^o^	Sex	Age (Years)	Cause of Injury	Injury Grade AAST	Type of Complication Size (mm^2^)	Treatment	Follow-Up
1	M	6	FH	III	Hematoma (20 × 37)	NOM	favorable
2	M	12	MVI	II	Hematoma (25 × 42)	NOM	favorable
3	M	11	FSH	III	Hematoma (23 × 52)	NOM	favorable
4	F	6	FSH	IV	Hematoma (34 × 24) + fever	NOM + AB	favorable
5	M	11	FH	III	Hematoma (56 × 41)	NOM	favorable
6	M	13	SI	III	Abscess (32 × 53) + fever	Punction + AB	favorable
7	M	15	FH	IV	Pseudocyst (33 × 55)	NOM	favorable
8	F	12	FSH	II	Hematoma (44 × 29)	NOM	favorable
9	F	6	MVI	III	Hematoma (43 × 21)	NOM	favorable
10	M	15	FI	IV	Hematoma (73 × 49) + fever	NOM + AB	favorable
11	F	17	FH	IV	Pseudocyst (20 × 25) + fever	NOM + AB	favorable
12	M	12	FB	I	Hematoma (22 × 31)	NOM	favorable
13	F	7	MVI	IV	Pseudoaneurysm with AV fistula (32nd day)	NOM	favorable
14	M	5	FSH	II	Hematoma (38 × 28)	NOM	favorable
15	M	5	FH	IV	Hematoma (59 × 23)	NOM	favorable
16	F	13	FI	IV	Pseudocyst (50 × 55)	Partial splenectomy	favorable
